# Case report: Metastatic ovarian mucinous carcinoma to the breast: diagnostic challenges and pitfalls

**DOI:** 10.3389/fonc.2024.1364011

**Published:** 2024-03-18

**Authors:** Natthawadee Laokulrath, Siew Kuan Lim, Hwee Yong Lim, Mihir Gudi, Puay Hoon Tan

**Affiliations:** ^1^ Department of Pathology, Siriraj Hospital, Mahidol University, Bangkok, Thailand; ^2^ Department of Pathology and Laboratory Medicine, Kandang Kerbau (KK) Women’s and Children’s Hospital, Singapore, Singapore; ^3^ Solis Breast Care and Surgery Centre, Singapore, Singapore; ^4^ Novena Cancer Centre, Singapore, Singapore; ^5^ Luma Medical Centre, Singapore, Singapore; ^6^ Parkway Laboratory Services Ltd, Singapore, Singapore

**Keywords:** metastatic to the breast, *in-situ*-like structures, basement membrane, nipple metastasis, ovarian mucinous carcinoma

## Abstract

Metastases to the breast from extramammary sources are extremely rare, with the ovary, primarily high-grade serous carcinoma, being the most common origin. We report a case of breast metastases from advanced stage ovarian mucinous carcinoma in a 48-year-old female— a case hitherto unreported in the literature. The case is noteworthy for its atypical presentation marked by an areolar rash, clinically suggestive of Paget disease of the nipple. This unique clinical scenario, coupled with histopathological examination revealing *in-situ*-like carcinoma component, posed a diagnostic challenge in discerning the tumour origin. We emphasize the need for heightened awareness among pathologists to avoid misdiagnosing metastatic carcinomas as primary breast tumours, a potential pitfall with significant clinical implications.

## 1 Introduction

Non-mammary metastatic carcinoma to the breast and axilla constitutes a rare subset, accounting for only 0.2-1.1% of all breast malignancies (1, 2), with haematologic metastases excluded. The gynaecologic tract is the most prevalent primary extramammary site, notably the ovary (3). High-grade ovarian serous carcinoma is the predominant type, followed by the much less common metastatic ovarian clear cell carcinoma (3).

Remarkably unusual, metastatic ovarian mucinous carcinoma to the breast has been scarcely documented, with only two related cases in the literature. One case involved seromucinous carcinoma (4), a subtype of endometrioid carcinoma according to the latest WHO classification of female genital tumours (5). Another patient had a mixed mucinous and mesonephric cystadenocarcinoma (6), where the breast biopsy exhibited a solely mesonephric appearance.

Herein, we present a distinct case of breast metastasis originating from advanced ovarian pure mucinous carcinoma, a scenario not previously documented. In this report, we delineate the intriguing histologic findings and discuss the diagnostic challenges inherent in such rare occurrences.

## 2 Case report

**Figure 1:**
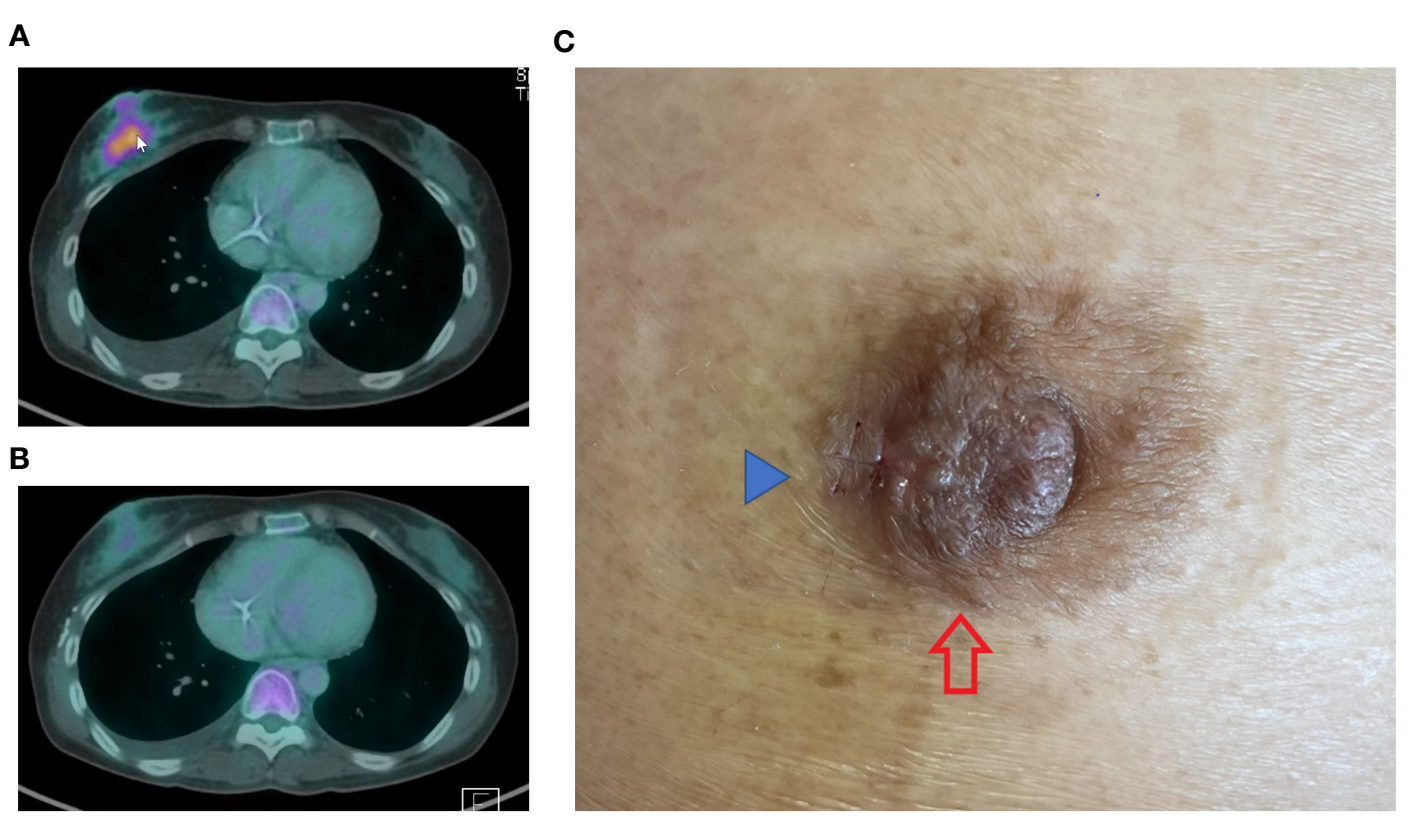
PET CT images dated 16/10/2023 **(A)** depict a highly metabolic right breast lesion. The cancer exhibited significant response following only 3 cycles of combination chemotherapy incorporating Trastuzumab (TCH regimen), as evidenced by the image from 18/12/2023 **(B)**. The nipple **(C)** showed a rash at the lateral aspect of the right nipple areolar complex (red arrow), clinically mimicking Paget disease. A small surgical scar (blue arrow) is observed at the lateral edge of the rash, attributed to a recent incisional skin biopsy.

A 48-year-old Chinese female with a previous diagnosis of mucinous ovarian cancer, initially identified in January 2022, experienced recurrence shortly after completing chemotherapy. The patient re-presented with extensive peritoneal disease, which showed a short duration response to second-line chemotherapy. Her disease progression was marked by rapid growth of two right breast masses, measuring 50 mm and 10 mm at 9 o’clock and 10 o’clock, respectively. Radiological assessments revealed interval detection of an FDG avid irregular lesion in the retro-areolar region of the right breast (size of 3.6cm x 2.3cm) (**Figure 1A**), with several new small mildly FDG avid satellite lesions seen in the rest of the right breast.

In addition to the breast masses, clinical examination detected a right areolar rash (**Figure 1C**). Core biopsies without prior fine needle aspiration cytology were performed on both masses, along with a nipple incisional biopsy.

Histological examination of the core biopsies from both masses revealed an invasive carcinoma characterized by dispersed nests, trabeculae, and tubules, comprising 10-75% of the tumour, infiltrating amidst benign and variably inflamed breast lobules and oedematous stroma. Predominantly, the tumour cells exhibited high-grade nuclei with abundant eosinophilic and foamy cytoplasm. Some regions displayed atypical glandular structures, partially lined by nuclei of relatively lower grade, featuring abundant basophilic apical mucin seamlessly transitioning into highly pleomorphic epithelial cells. Notably, no extracellular mucin, cystic-papillary structures, conspicuous goblet cell, or signet ring morphologies were observed. There were dense collagenous bands encircling a few glands, resembling intact basement membranes. Adjacent to these bands were inconspicuous, stretched-out, and compressed cells, akin to myoepithelial cells. Lymphovascular invasion was evident in three foci within one section of the breast core obtained from the 9 o’clock mass, while its presence in the 10 o’clock mass was equivocal. Additionally, the 9 o’clock lesion exhibited a fibroadenoma with carcinoma involvement. Non-neoplastic breast tissue revealed adenosis accompanied by chronic inflammation.

**Figure 2:**
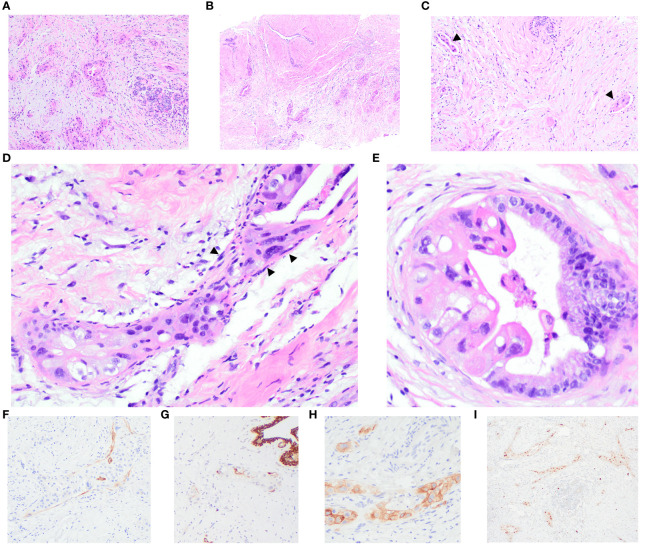
Breast core biopsies illustrate the invasive carcinoma, showing perilobular infiltration **(A)** and involvement of a fibroadenoma **(B)**. Multiple foci of lymphovascular invasion are evident (arrowheads) **(C)**. Distinctive features include dense collagenous bands encircling some glands, resembling a basement membrane with stretched-out and compressed cells (arrowheads), akin to myoepithelial cells **(D)**. A few glands are partially lined by columnar cells with lower-grade nuclei and abundant basophilic apical mucin, transitioning seamlessly into highly pleomorphic epithelial cells **(E)**. IHC results demonstrate scattered positivity for smooth muscle myosin heavy chain (SMMHC) **(F)** and cytokeratin 5 (CK5) **(G)** around the tumour nests. Carcinoma cells display positive staining for CA125 **(H)** and PAX8 **(I)**.

Immunohistochemistry (IHC) analysis showed diffuse positive reactivity for PAX 8 in the nuclei of the malignant cells, while GATA3, GCDFP15, and WT1 exhibited negative staining. Positive CA125 reactivity was observed in the malignant glands. Smooth muscle myosin heavy chain (SMMHC) revealed general negativity around malignant glands and tumour nests, with scattered peripheral positivity around some malignant groups. CK5 highlighted scattered malignant cells, with some tumour nests showing apparent peripheral positive decoration. p63 was negative around tumour nests and malignant glands. Mammaglobin displayed patchy faint to weak cytoplasmic blush in occasional malignant cells. ER and PR were negative, evidenced by no staining, with optimally stained normal breast epithelium. Equivocal HER2 IHC staining (score 2+) was noted in 30% and 40% of tumour cells in the 9 o’clock and 10 o’clock lesions, respectively, displaying weak-to-moderate intensity. VENTANA HER2 Dual ISH DNA Probe cocktail showed positive results (group 1) for *HER2* gene amplification; the average HER2:CEP17 ratios were 2.7 and 3.2 in the 9’clock and 10 o’clock tumours, respectively. A detailed representation of the histology and immunohistochemical studies of the core biopsies of the breast masses is provided in **Figure 2**.

**Figure 3:**
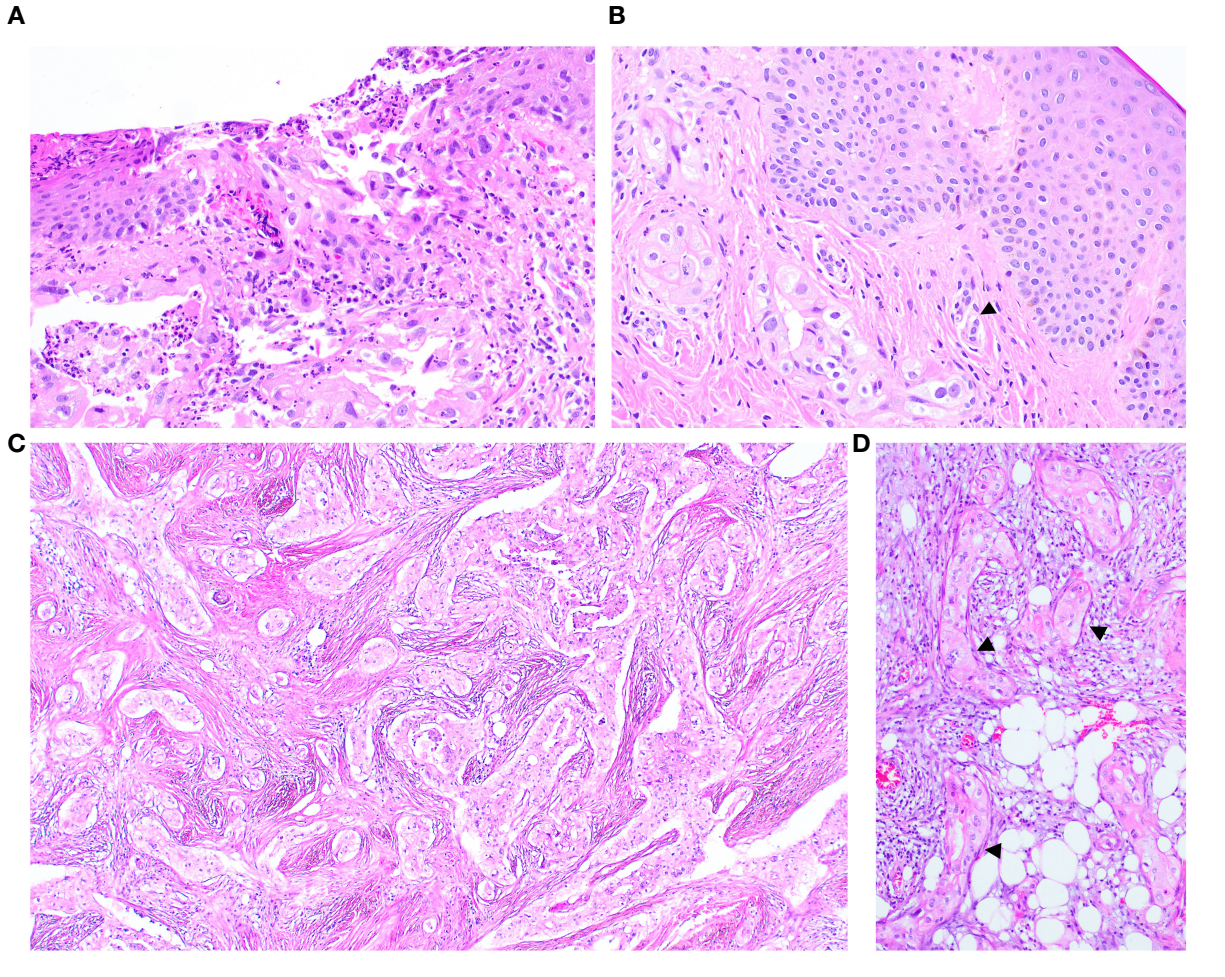
The nipple biopsy reveals infiltrating carcinoma involving the epidermis with focal epidermal erosion **(A)**, extending into the dermis with dermal lymphovascular invasion (arrow) **(B)**. The metastatic carcinoma in the omentum shows a comparable morphology to the tumour present in the breast **(C)** an *in-situ*-like structure (arrow) is also identified **(D)**.

The nipple biopsy section demonstrated malignant glands and nests with identical histomorphology to the carcinomas described above. The infiltrating carcinoma involved the epidermis, dermis, and deeper parts of the nipple stroma and areolar muscle. Focal epidermal erosion was identified, though the epidermis was devoid of abnormal intraepidermal epithelial clusters. Several malignant cells exhibited cytoplasmic mucinous vacuoles, and dermal lymphovascular invasion was present (**Figure 3**).

The histology of the primary ovarian tumour was unavailable for review. Subsequent biopsies, displaying identical appearances, revealed multiple metastases to the omentum - the largest tumour deposit being 5 cm, pelvic peritoneum, bilateral sides of the diaphragm, liver capsule and gallbladder bed, including to the serosa, muscularis propria and mucosa of the sigmoid colon. The basement membrane-like matrix with occasional basal stretched-out cells were also noted in the section of metastatic carcinoma to the omentum and pelvic peritoneum (**Figure 3B**).

Upon identification of a positive (HER2) Dual *In Situ* Hybridization (DISH) result in both the breast and peritoneal specimens, the clinical chemotherapeutic regimen was revised to a more manageable and well-tolerated combination with an anti-HER2 drug. The tumour exhibited a remarkable and positive response to the modified chemotherapeutic regimen (**Figure 1B**).

## 3 Discussion

We present an unusual case of metastatic ovarian mucinous carcinoma, clinically presenting as breast masses and a nipple rash mimicking Paget disease of the nipple. Histologically, the carcinoma exhibited infiltration into periductal and perilobular areas, sparing terminal duct-lobular structures, and involving a fibroadenoma. Notably, the carcinoma displayed mucinous features with high-grade cytomorphology, lacking extracellular mucin and the characteristic appearance of mucinous cystadenocarcinoma of the breast, specifically the absence of cystic areas with papillary epithelial proliferation.

Distinctive features included a subset of glands partially lined by tall columnar cells exhibiting relatively lower grade nuclei and abundant basophilic intracytoplasmic mucin, reminiscent of Mullerian mucinous epithelium. These features deviate from the typical morphology of invasive mammary carcinoma. Considering the patient’s history of ovarian mucinous carcinoma (which was not initially available), the diagnosis of metastatic carcinoma was favoured. However, the mucinous differentiation was only focally present and it is acknowledged that the tumour appearance may be influenced by chemotherapy, and the biopsy samples may not fully capture the heterogeneous morphology of the tumour. Consequently, the possibility of a primary breast carcinoma could not be definitively ruled out.

The presence of a basement membrane-like matrix encircling tumour nests and glands, coupled with occasional juxtaposition of stretched-out, flat to oval darker-stained nuclei, prompted consideration of *in-situ* carcinoma and a primary tumour. However, immunohistochemical staining for myoepithelial cells revealed mostly equivocal positivity though some accentuation of peripheral staining could be potentially interpreted as reflecting attenuated myoepithelial cells. Interestingly, upon comparing the histology with that of biopsies from other metastatic sites in subsequent specimens, the *in situ*-like pattern was also observed in the pelvic peritoneal sections, refuting the notion of a genuine *in-situ* process for this appearance.

Immunohistochemically, the carcinoma cells exhibited diffuse positivity for PAX8 and CA125, while testing negative for breast immunomarkers such as GATA3 and GCDFP15. The combined histologic findings and immunoprofile supported a metastatic ovarian origin.

Literature reviews underscore the rarity of metastatic extramammary carcinomas to the breast, with only a small proportion (11%) (3) presenting with breast or axillary lesions as the initial manifestation, while the majority (77%) (3) already have disseminated disease upon breast metastasis detection. Clinical history proves pivotal for accurate diagnosis, but in instances where information is lacking or inaccessible, non-mammary metastases can be challenging to identify and may be misdiagnosed as primary breast cancer.

The patients’ age ranges from 15 to 83 years, with a median age of 54 years (3). Tumour sizes exhibit a median of 1.68 cm, ranging from 0.5 to 18 cm (3). Radiologically, metastatic tumours often lack specific features but are typically unilateral, singular masses, and frequently located in the upper outer quadrant, accounting for 50-60% of cases (7, 8). These findings may mimic benign and malignant breast tumours, adding complexity to the diagnostic process.

Histologically, metastatic tumours in the breast can disclose various patterns (7, 9, 10), including a circumscribed tumour, which is the most prevalent, featuring a well-defined mass surrounded by normal breast tissue. In some cases, the tumours form nodules distributed around ducts and lobules. Another pattern involves lymphangitis carcinomatosis, where multiple dispersed tumour clusters are present within dilated lymphatic spaces. In addition, a diffuse involvement of breast parenchyma may occur, indicating a more widespread infiltration of tumour cells throughout the breast tissue.

Microscopic findings identified in prior studies indicative of metastasis include features that are unusual for breast carcinoma (1, 3, 11, 12), the absence of *in-situ* carcinoma (1, 3, 11, 12), a well-circumscribed or pushing tumour border enveloped by a fibrous pseudocapsule (3, 11), the lack of elastosis (1, 11, 12), and the presence of multiple satellite foci (11). While lymphatic emboli are recognized as suggestive of metastatic disease (1, 12), it is noteworthy that lymphovascular invasion was found to be absent in 87% of cases in one study (3).

The presence of an intraductal component of carcinoma is consistently noted in the literature as supportive evidence for primary breast cancer (1, 3). However, we highlight the potential diagnostic pitfall of relying solely on *in-situ* appearances to support the diagnosis of primary breast carcinoma. *In situ*-like metastatic foci that mimic *in-situ* mammary carcinoma have been occasionally reported in the literature, offering two plausible explanations for this phenomenon. The first scenario involves the spread of metastatic ovarian cancer cells into existing mammary ducts, as illustrated by Maeshima Y. et al. (4), where the *in situ*-appearing architecture exhibited neoplastic cells having the same morphology as metastatic seromucinous carcinoma, surrounded by confirmed myoepithelium. A similar finding is described in metastatic colonic adenocarcinoma to the biliary tract, where intraepithelial growth mimics primary intrabiliary carcinoma (13). The second scenario is lymphovascular invasion mimicking *in situ* disease, proposed by Gupta D et al. (14), involving metastatic renal cell carcinoma and metastatic ovarian papillary serous adenocarcinoma. These cases showed multiple tumour emboli floating within and plugging lymphatic spaces. In some foci, metastatic carcinoma cells adhered to the endothelium and expanded the lymphatic spaces, mimicking ductal carcinoma *in situ*. Conclusive evidence was provided by immunohistochemically highlighting the endothelium with CD31, CD34, and Ulex europaeus, observing adjacent vascular structures with accompanying extensive lymphovascular invasion. Also noted in the study was desmoplastic and inflammatory response around dilated lymphatic spaces and necrosis within the tumour clusters in lymphatic spaces mimicking periductal stromal change and comedonecrosis seen in ductal carcinoma *in situ*, respectively (14).

To differentiate DCIS from tumour emboli, myoepithelial and endothelial immunomarkers should be considered. Caution is warranted in the evaluation of myoepithelial immunohistochemical (IHC) markers, as 84.2% (15) of ductal carcinoma *in situ* (DCIS) cases have demonstrated diminished IHC expression in myoepithelial cells, particularly in high-grade DCIS. Of note, smooth muscle myosin heavy chain (SMMHC) exhibits significantly reduced reactivity in these cases. In this context, SMA, p75, p63, and calponin may offer greater sensitivity and may be preferable for assessing myoepithelial cells (15). Additionally, the expression of D2-40, commonly employed for detecting lymphovascular invasion, has been observed in varying degrees in myoepithelial cells in mammary carcinoma *in situ* (16).

The encircling collagenous band around epithelial nests may resemble a native basement membrane. Such basal-membrane-like structures have also been documented in breast carcinoma metastasis to the lymph nodes (17) and many types of malignant tumours, for example, basaloid squamous carcinoma of the gastrointestinal tract (18) and pancreas (19), and invasive basal cell carcinoma of the skin (20). In the context of breast carcinoma, the presence of *in-situ*-like structures in metastatic sites supports their being reactive stroma rather than an *in-situ* process (21).

In our case, immunohistochemical stains for myoepithelial cells (SMMHC, CK5 and p63) produced equivocal results with some suggestion of focal positive rimming of occasional malignant nests. The existence of multiple foci of lymphovascular invasion and the proximity of small vessels adjacent to the *in-situ*-like foci raise the possibility of tumour emboli mimicking carcinoma *in situ*. Additionally, the basement membrane-like structures were identified in extramammary tumours (pelvic peritoneum), supporting metastatic disease.

Therefore, reviewing the histology of the prior malignancy and other synchronous tumours might assist in the diagnosis, as exemplified in our case. The likelihood of a diagnosis of metastatic carcinoma is strengthened if there is similar morphology to the prior carcinoma.

Immunohistochemical stains can be valuable in identifying the primary site of the tumour; however, they can also introduce complexity into the diagnosis, particularly in cases of mucinous-type ovarian carcinoma. This subtype tends to exhibit a divergent immunohistochemical profile from the typical pattern (CK7+/CK20-/PAX8+) observed in other epithelial-type ovarian tumours. CK7 and CK20 show varied positivity in ovarian mucinous tumours, with the majority displaying positivity for CK7 (22). The staining variability observed poses a challenge in differentiating ovarian mucinous tumours from primary breast carcinoma. The immunoprofile of CK7 positivity and CK20 negativity is akin to that of breast carcinoma, while addition of PAX8 positivity aligns more with an ovarian origin.

However, PAX8, a Mullerian immunomarker, is negative in 80-90% of ovarian mucinous carcinomas (23, 24). Similarly, SOX17, identified as a novel and promising biomarker with high specificity for gynaecologic tumours, produced positive results in only 23% of ovarian mucinous carcinomas (25). It is noteworthy that PAX8 positivity with variable staining intensity and tumour percentage is also observed in 6.02% of invasive mammary carcinoma, mostly high-grade with triple-negativity (26).

WT1 is not contributory in distinguishing between ovarian mucinous and breast carcinoma, as both can be negative (23). This observation is supported by Nonaka D et al. (23), in their study, where they found that WT1 expression was observed in 64% of pure and 33% of mixed mucinous breast carcinomas. The expression of WT1 was usually weak and focal in most of the positively staining breast tumours (3).

CA125 proves to be helpful in this context, as 90% of ovarian carcinomas are positive for CA125, exhibiting strong and diffuse staining, while the majority of breast carcinomas are negative for CA125 (27). Only 16% of primary and 12% of metastatic breast carcinomas showed weak and focal positivity (27). Mucinous cystadenocarcinoma of the breast has also been reported to be negative for CA125, although data on this entity are limited due to its rarity (28).

Breast immunomarkers prove highly valuable in this situation, as the majority of ovarian carcinomas were negative for these markers (29–31). It is important to note that a subset of ovarian mucinous carcinomas (2 out of 20 cases in one study (29)) can be positive for GATA3 (29), and 4% of ovarian tumours can express GCDFP-15 as well (2). TRPS1 appears to have higher sensitivity and specificity than GATA3 (31). Therefore, using TRPS1 immunohistochemistry as an adjunct with traditional breast and other markers may confirm or exclude a breast origin. However, 8% of ovarian non-serous carcinoma showed variable positivity for TRPS1 (31).

In our case, positivity for PAX8 and CA125 and negativity of markers associated with a breast origin supported ovarian metastasis. The metastatic carcinoma also exhibited HER2 amplification. HER2 overexpression has been documented in 25-40% of ovarian mucinous carcinomas (32, 33). Therefore, pathologists should exercise caution and not be misled by a positive HER2 result as supportive evidence for primary breast origin.

Nipple involvement by metastatic carcinoma is an exceedingly uncommon occurrence. Our case represents, to our knowledge, the first reported instance of nipple involvement by metastatic ovarian carcinoma, clinically manifesting as an areolar rash and mimicking Paget disease of the nipple. Histological examination revealed epidermal erosion with tumour involvement, albeit without the presence of intraepidermal tumour nests. While the literature review identified a limited number of cases depicting metastatic ovarian high-grade serous carcinoma and clear cell carcinoma to the breast, simulating inflammatory breast carcinoma, and an ovarian serous carcinoma metastases to an intramammary lymph node mimicking a primary breast carcinoma (34–38), such a clinical presentation was not evident in our cases. Furthermore, the antecedent case reports did not report nipple involvement.

The identification of a metastatic tumour within the breast commonly heralds an unfavourable prognosis, as a substantial proportion of patients already manifest widespread disease. According to a case series (3), mortality was observed in 96% of patients with available follow-up data, culminating in a median survival period of 15 months subsequent to the diagnosis of the breast or axillary lesion.

In summary, we highlight the crucial importance of accurate diagnosis when dealing with these tumours to avoid unnecessary surgical procedures or treatments. The case presented emphasizes a diagnostic strategy that focuses on identifying morphology favouring metastatic carcinoma, particularly considering the patient’s history of extramammary malignancy and the unusual histology that does not align with primary breast cancer. The identification of carcinoma *in-situ*-like foci, while conventionally indicative of a breast primary, introduces a potential diagnostic pitfall. Awareness of mimics, such as a basement membrane-like matrix or *in-situ*-like structures signifying the dissemination of metastatic cancer cells into pre-existing mammary ducts and lymphatic emboli, is crucial. Consequently, the *in-situ* appearance should not be construed as conclusive pathognomonic evidence of primary disease unless complemented by additional histologic and immunohistochemical support. Comparing tumour histology with specimens from primary and metastatic sites refines diagnosis. Tailoring immunohistochemical stains based on the patient’s non-breast malignancy history and carcinoma morphology is crucial. A broad immunohistochemical panel, including multiple organ-specific markers for potential origins, is imperative to avoid pitfalls in interpretation.

## Data availability statement

The original contributions presented in the study are included in the article/supplementary material. Further inquiries can be directed to the corresponding author.

## Ethics statement

Ethical approval was not required for the studies involving humans because this is a case report and it does not require an ethics committee review. The studies were conducted in accordance with the local legislation and institutional requirements. Written informed consent was obtained from the individual(s) for the publication of any potentially identifiable images or data included in this article.

## Author contributions

NL: Writing – original draft, Writing – review & editing. SL: Resources, Writing – review & editing. HL: Resources, Writing – review & editing. MG: Writing – original draft, Writing – review & editing. PT: Writing – original draft, Writing – review & editing.
